# Adipose tissue‐derived extracellular vesicles aggravate temporomandibular joint osteoarthritis associated with obesity

**DOI:** 10.1002/ctm2.70029

**Published:** 2024-09-30

**Authors:** Baochao Li, Yuqin Jin, Bingqing Zhang, Tong Lu, Jialing Li, Jingzi Zhang, Yiwen Zhou, Yanyi Wang, Caixia Zhang, Yue Zhao, Huang Li

**Affiliations:** ^1^ Nanjing Stomatological Hospital Affiliated Hospital of Medical School, Nanjing University Nanjing China; ^2^ The State Key Laboratory of Pharmaceutical Biotechnology, Division of Immunology Medical School, Nanjing University Nanjing China

**Keywords:** adipose tissue‐derived extracellular vesicles, geranylgeranyl diphosphate synthase, miR‐3074‐5p, temporomandibular joint osteoarthritis

## Abstract

**Introduction:**

Temporomandibular joint osteoarthritis (TMJ OA) is a major disease that affects maxillofacial health and is characterised by cartilage degeneration and subchondral bone remodelling. Obesity is associated with the exacerbation of pathological manifestations of TMJ OA. However, the underlying mechanism between adipose tissue and the TMJ axis remains limited.

**Objectives:**

To evaluate the effects of obesity and the adipose tissue on the development of TMJ OA.

**Methods:**

The obesity‐related metabolic changes in TMJ OA patients were detected by physical signs and plasma metabolites. The effects of adipose tissue‐derived EVs (Ad‐EVs) on TMJ OA was investigated through histological and cytological experiments as well as gene editing technology. Alterations of Ad‐EVs in obese state were identified by microRNA‐seq analysis and the mechanism by which EVs affect TMJ OA was explored in vitro and in vivo.

**Results:**

Obesity and the related metabolic changes were important influencing factors for TMJ OA. Ad‐EVs from obese mice induced marked chondrocyte apoptosis, cartilage matrix degradation and subchondral bone remodelling, which exacerbated the development of TMJ OA. Depletion of Ad‐EVs secretion by knocking out the geranylgeranyl diphosphate synthase (*Ggpps*) gene in adipose tissue significantly inhibited the obesity‐induced aggravation of TMJ OA. MiR‐3074‐5p played an important role in this process .

**Conclusions:**

Our work unveils an unknown link between obese adipose tissue and TMJ OA. Targeting the Ad‐EVs and the miR‐3074‐5p may represent a promising therapeutic strategy for obesity‐related TMJ OA.

**Key points:**

- High‐fat‐diet‐induced obesity aggravate the progression of TMJ OA in mice.
- Obese adipose tissue participates in cartilage damage through the altered miRNA in extracellular vesicles.
- Inhibition of miR‐3074‐5p/SMAD4 pathway in chondrocyte alleviated the effect of HFD‐EVs on TMJ OA.

## Graphical abstract

1. High‐fat‐diet‐induced obesity aggravate the progression of TMJ OA in mice.
2. Obese adipose tissue participates in cartilage damage through the altered miRNA in extracellular vesicles.
3. Inhibition of miR‐3074‐5p/SMAD4 pathway in chondrocyte alleviated the effect of HFD‐EVs on TMJ OA.

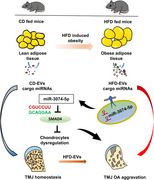

## 1 INTRODUCTION

Temporomandibular joint osteoarthritis (TMJ OA) is a nonbacterial inflammatory lesion and its main pathological indications include articular cartilage degeneration and subchondral bone remodelling.^1^, ^2^ Various elements including mechanical stress, hormonal influences, aging and psychological conditions are associated with TMJ OA, yet the precise causative factors are still not well defined.^3^

Recent studies have shown that lipid metabolism dysregulation is an established risk factor for TMJ OA.^4^ The influence of obesity on osteoarthritis has attracted widespread attention. In addition to promoting osteoarthritis in weight‐bearing joints such as the knee joint by increasing the mechanical load on the joints, obesity affects the lesions of non‐weight‐bearing joints through metabolic pathways.^5^, ^6^, ^7^ The biochemical properties of TMJ and knee joints are different. Knee cartilage is hyaline cartilage with hyaluronic acid as the main structure; in contrast, the surface of the mandibular condyle is fibrocartilage rich in type II collagen.^8^, ^9^ Adipose tissue, functioning as a dynamic endocrine organ, emits numerous adipokines that have multiple roles in the development of osteoarthritis in the knee joint.^10^, ^11^ Clinical investigations have revealed a correlation between temporomandibular joint disorders and the degree of obesity,^12^, ^13^, ^14^, ^15^ indicating that the role of adipose tissue and metabolism in temporomandibular joint should not be underestimated. However, further information on the relationship between adipose tissue and the TMJ axis remains limited.

Extracellular vesicles (EVs) generally encapsulate a range of bioactive components including microRNAs (miRNAs) and proteins, participating in numerous pathological or physiological processes.^16^, ^17^ EVs derived from adipose tissue (Ad‐EVs) contribute to the advancement of multiple diseases, including osteoporosis and cognitive deficits.^18^, ^19^ However, whether Ad‐EVs in obese individuals are related to the pathogenesis of TMJ OA has not been studied. When an individual becomes obese, the number and function of Ad‐EVs change. It has been reported that obese mice secrete twice as many EVs per unit of adipose tissue as normal mice.^20^ Not only that, the miRNA expression profile of Ad‐EVs in obese individuals is altered and these miRNAs may play important roles metabolic disorders.^21^ Given that the functions of miRNAs in the pathogenesis of osteoarthritis remains unclear,^22^ more efforts are needed to study the relationship between obesity‐associated EV miRNAs and TMJ OA.

In this study, we found that obesity significantly aggravated mechanical force‐related TMJ OA. Notably, Ad‐EVs from obese mice and their miR‐3074‐5p cargo induced marked cartilage matrix degradation and subchondral bone remodelling in TMJ. These results elucidate a new metabolic signalling axis between adipose tissue and the TMJ, potentially revealing a new therapeutic target of TMJ OA. Targeting the Ad‐EVs and its miR‐3074‐5p may represent a promising therapeutic strategy for obesity‐related TMJ OA.

## 2 METHODS

### 2.1 Human information

All human information and samples were obtained from Nanjing Stomatological Hospital. Both healthy controls and TMJ OA patients expressed readiness to take part in this research. Patients were enrolled based on the diagnostic criteria for osteoarthritis/osteoarthrosis classification in the *Research Diagnostic Criteria for Temporomandibular Disorders*.^23^ Patients were excluded if they had the following conditions: (1) treatment history of temporomandibular joint disease; (2) temporomandibular joint injury caused by trauma, tumour and so on; (3) systemic disease; (4) autoimmune disease; (5) pregnancy. The weight and height of the patients were recorded and their blood samples were collected before treatment. For the detection BMI values (weight (kg)/height(m)^2^), a total of 64 TMJ OA patients (23 males and 41 females) and 75 healthy controls (37 males and 38 females) were incorporated. The experiments involving plasma procurement involved 28 TMJ OA patients and 29 healthy controls. The Ethics Committee of Nanjing Stomatological Hospital (NJSH‐2022NL‐39) sanctioned this study, and all participants provided written informed consent.

### 2.2 Lipidomic assays

The plasma concentration of free fatty acids (FFA) was ascertained using high‐performance liquid chromatography–mass spectrometry (HPLC–MS).^24^, ^25^ Briefly, 50 μL plasma was vortexed with 225 μL methanol for 10 s, followed by the addition of 750 μL of MTBE and agitation for 10 min at 4°C. Subsequently, 188 μL of ultrapure water was incorporated, stirred for 20 s, and spun at 14 000 rpm for 2 min at 4°C. We extracted 350 μL of supernatant and situated it in a centrifuge for desiccation. The desiccated samples were reconstituted with 110 μL of compound solution, spun at 18 000 rpm for 10 min. The supernatant was separated and detected. The MS‐DIAL software (version 4.80) was used to identify and quantify different types of fatty acids in plasma.

### 2.3 Animal models

C57BL/6J male mice were provided by the Model Animal Research Center of Nanjing University. Adipose‐specific geranylgeranyl diphosphate synthase (*Ggpps*) knockout mice (cKO) were generated by breeding Adiponectin‐Cre mice with *Ggpps*‐floxed transgenic mice. All mice were housed in SPF facility with three mice per cage. The feeding environment was stably controlled at (22 ± 2)°C, humidity 45−55%. All mice were nourished with a chow diet (CD) containing 10% of fat (SWS9102; Xie‐Tong, Nanjing, China) or high‐fat diet (HFD) containing 60% of fat (XTHF60; Xie‐Tong, Nanjing, China). For the obese group, WT and *Ggpps* cKO mice were fed with HFD for 12 weeks.^26^

For TMJ OA group, WT and Ggpps cKO mice were treated with unilateral anterior crossbite models (UAC), which is a classic method to establish TMJ OA in mice or rats.^27^, ^28^, ^29^ Briefly, when each mouse was anaesthetised using pentobarbital (40 μL/10 g body weight), a short metal tube (1.5 mm long, .61 mm inner diameter) and another long tube (4.5 mm long, .61 mm inner diameter) were attached to its left maxillary and mandibular incisor. The top 1 mm of the mandibular tube was bent a 45° labial angle. Mice in the relevant groups started the UAC model at 10 weeks of age and continued for 8 weeks. The metal tubes were checked every other day to ensure that the detached tubes could be re‐bonded. All of the protocols were endorsed by the Animal Care and Use Committee of Nanjing University (IACUC‐D2204005).

### 2.4 Micro‐CT

Mice were euthanised at 18 weeks and the TMJ tissues were collected. The joints were scanned by Micro‐CT scanner (Skyscan 1176; Belgium) at 50 kV, 400 μA and 6.0 μm per pixel. As we and other teams previously described,^30^, ^31^ a cubic section (.4 × .4 × .4 mm) at the centre of the subchondral bone were selected as the region of interest (Figure S1). The microstructural parameters including BV/TV (bone volume per tissue volume), Tb.Th (trabecular thickness), Tb.N (trabecular number) and Tb.Sp (trabecular separation) were measured by CTAn software (Bruker microCT; Germany) with the threshold of 80−255. The Micro‐CT analysis was conducted by two technicians who were blinded to the study and then collated by an author blinded to the groups of the samples.

### 2.5 Histological and immunohistochemical analyses

The TMJ specimens were fixed 24 h in 4% paraformaldehyde and then decalcified at EDTA (10%; Sigma–Aldrich) for 4 weeks. The decalcified TMJ tissues were embedded in paraffin and then cut into 5 mm‐thick slices along the sagittal plane. Staining of the slices was performed using haematoxylin–eosin (H&E) and Safranine O‐Fast Green (SO) staining as previously reported.^32^ The pathological changes of articular cartilage were quantified according to the modified Mankin's scoring criteria.^33^, ^34^

Immunohistochemistry was performed according to avidin–biotin complex staining methods. Briefly, the slices were sequentially soaked in xylene and serial alcohol solutions to remove paraffin. Following antigen retrieval, 3% hydrogen peroxide was utilised to diminish peroxidase activity. Specimens then underwent 5% goat serum to minimise nonspecific staining before being incubated with antibodies. The antibodies were identified by treatment with DAB substrate. Slices were dehydrated in serial alcohol solutions and xylene and then sealed with resin.

### 2.6 ATDC5 cells culture study

ATDC5 cells serve as an in vitro model accurately mimicking the intricate differentiation phases of chondrocytes under consistent culture conditions.^35^, ^36^ The ATDC5 cells were cultured in DMEM/F12 medium (5% foetal bovine serum, 1% penicillin/streptomycin) in 5% CO_2_ at 37°C. In all vitro experiment, the chondrocytes were cultured to the P2–P3 generation and then treated according to different groups. For chondrogenic differentiation induction, the culture conditions were switched to differentiation medium (growth medium with ITS [5 μg/mL human transferrin, 10 μg/mL insulin, 30 nM sodium selenite]) and the medium was changed every alternate day. The chondrocytes were able to synthesise proteoglycans and remained chondrogenic in phenotype in their in vitro cultures (Figure S2). For the following experiments, chondrocytes were digested with trypsin, resuspended and then seeded into six‐well with 1 × 10^5^ cells/well or 12‐well plates with 5 × 10^4^ cells/well. To observe the effect of Ad‐EVs on chondrocyte viability, the cells were stimulated with either 10 ng/mL IL‐1β or 5 μg/mL EVs for 24 h and then subjected to subsequent assays.^1^, ^37^, ^38^

For co‐culture experiment, the murine ATDC5 chondrocytes were cultured in 12‐well plates with 5 × 10^4^ cells/well. Visceral adipose tissues of lean and obese mice were cut into pieces and cultured in a transwell system (.4 mm) which was placed above the chondrocytes. To limit the secretion of EVs from adipose tissue, we pretreated adipose tissue with GW4869 at a concentration of 10 μM for 24 h prior to co‐culture with chondrocytes.^39^

### 2.7 Isolation and characterisation of Ad‐EVs

After the mice were sacrificed at 18 weeks of age, Ad‐EVs from lean mice and obese mice were extracted as previously reported.^40^ In brief, visceral adipose tissue from mice was washed in PBS and cut into pieces smaller than .1 cm^3^. The adipose fragments were then incubated in exosome‐free medium for 24 h. The media were spun at 1000×*g* for 5 min and 3000×*g* for 10 min; then spun at 10 000×*g* for 30 min. The supernatant was finally spun at 110 000×*g* for 70 min and the precipitate was then resuspended using PBS. This procedure was duplicated, and the sediment was redissolved in 200 μL PBS to obtain Ad‐EVs.

### 2.8 Transmission electron microscope analysis

The Ad‐EVs solution was fixed for 10 min using equal volume of 4% paraformaldehyde. Then, the Ad‐EVs were stained chemically by adding 4% uranyl acetate, and the resulting images of EVs morphology were acquired using transmission electron microscope (Hitachi, Japan).

### 2.9 Nanoparticle tracking analysis

The concentration and particle size distribution of Ad‐EVs were tested by NanoSight (Malvern, UK). Briefly, the EVs were diluted in PBS at a fixed ratio and then added to the ZetaView. Upon completion of the detection, information regarding particle concentration, size and concentration distribution was acquired. Each group underwent analysis in three replicates.

### 2.10 Western blot detection

Proteins were isolated using RIPA lysis buffer (Beyotime, Shanghai) by spin at 12 000 rpm for 15 min. The protein concentration of supernatant was ascertained employing the BCA kit (Beyotime). Protein samples underwent separation through gel electrophoresis and were subsequently electroblotted onto a PVDF membrane, followed by blocking with 5% non‐fat milk. The proteins were combined with primary antibodies overnight at 4°C. After washing with TBST for 3 × 10 min, secondary antibodies were administered. Chemiluminescence signals were detected using BeyoECL Plus (Beyotime). Details of the antibodies involved in this experiment are given in Table S1.

### 2.11 Tracking Ad‐EVs in vivo

EVs were stained with 1 μM DiR (Umbio, China) or DiO (Invitrogen, America) according to the instructions. For in vivo tracking, 100 μL of DiR/DiO‐labelled EVs (1 μg/μL) were administered to mice through the tail vein. Fluorescence of DiR‐labelled EVs in mice was captured using the IVIS Spectrum system 24 h after injection (Caliper Life Sciences, USA). TMJ were also frozen‐sectioned and later stained using DAPI for visualising the distribution of DiO‐labelled EVs through confocal microscopy analysis.

### 2.12 Tracking Ad‐EVs in vitro

EVs were stained with 100 μM PKH26 (UR52302; Umbio) according to the instructions. For in vitro tracking, chondrocytes were incubated with PKH26‐labelled EVs (5 μg/mL) for 12 h. Then, chondrocytes were observed under a fluorescence microscope (NIKON Ti, Japan) followed by treatment with FITC‐labelled phalloidin and DAPI (Santa Cruz, USA).

### 2.13 Scratch assay

The scratch assay served to assess the proliferation and migration capabilities of chondrocytes. In brief, chondrocytes were cultured in six‐well plates separated by a rubber baffle and the baffle was removed when the cells reached 100% confluence, creating a cell‐free band of approximately 100 μm. After washing with PBS, the images of each treatment group were recorded by microscope (Nikon TS2) at 0, 12 and 24 h after scratch formation.

### 2.14 Flow cytometry assay

Chondrocytes from the different treatment groups were resuspended and counted. After resuspending in binding buffer, the chondrocytes underwent incubation with PI and Annexin V‐FITC (Vazyme Biotech, Nanjing) in a dark environment for 20 min. Apoptosis rate was then counted using flow cytometry.

### 2.15 MiRNA expression analysis

MiRNA of Ad‐EVs from WT mice on CD or HFD and cKO mice on HFD was extracted using Trizol. There were three experimental replicates in each group. The samples were quantified using the Agilent Bioanalyzer assay kit (Novogene, Beijing). Library preparation was conducted following the guidelines of the Illumina kitas previously described.^41^ The heatmap of miRNA was established using the mean value of miRNA levels in several groups of EVs.

### 2.16 qRT‐PCR

Total mRNA/miRNAs were extracted by RNAiso (Vazyme Biotech) adhering to the guidelines. For mRNA reverse transcription, we mixed 4 μL of cDNA synthesis mix (Vazyme Biotech) with 1000 ng of mRNA from each sample. For miRNA reverse transcription, looped miRNA‐specific primers were used with a miRNA cDNA synthesis kit (Vazyme Biotech). The PCR reaction was generated at ABI Viia 7 (Thermo) using ChamQ Universal SYBR qPCR Mix (Vazyme, Nanjing). ΔΔCT method was applied for results calculation with normalisation to GAPDH (for mRNA) or U6 (for miRNA). Details of the primer sequences are detailed in Table S2.

### 2.17 Cell transfection

Transfection was performed by Lipofectamine 2000 (Thermo) when chondrocytes had grown to 60−70% confluence. The negative control and inhibitor of miR‐3074‐5p (Keygen Biotech) were transfected at 100 nM for 6 h. Normal medium was replaced to terminate transfection subsequently. Similarly, the overexpression plasmids of SMAD4 were constructed using the GV657‐puro vector (Genechem Biotech) and transfected at 50 nM.

### 2.18 Luciferase assays

The report gene that matched SMAD4 mRNA sequence and possessed the binding sequence of miR‐3074‐5p (wild‐type [WT] or mutant [MUT]) was constructed by Genechem (Nanjing, China). Chondrocytes were cultured to 60−70% confluence and then co‐transfected with psiCHECK™‐1‐UTR and miR‐3074‐5p (mimic or NC). Twenty‐four hours later, the chondrocytes were lysed for luciferase activity detection using the Dual‐Luciferase reporter system (Vazyme Biotech).

### 2.19 Statistical analysis

Statistical analysis of the data was conducted using GraphPad Prism 8.0 (San Diego, CA, USA). The *p* value was calculated using the Student's *t*‐test for two groups and ANOVA for multiple groups, with significance set at *p* < .05. Statistical analyses were carried out on data from at least three independent experiments, and quantitative results were presented as mean ± standard error.

## 3 RESULTS

### 3.1 Obesity exacerbates the manifestations of TMJ OA

**FIGURE 1:**
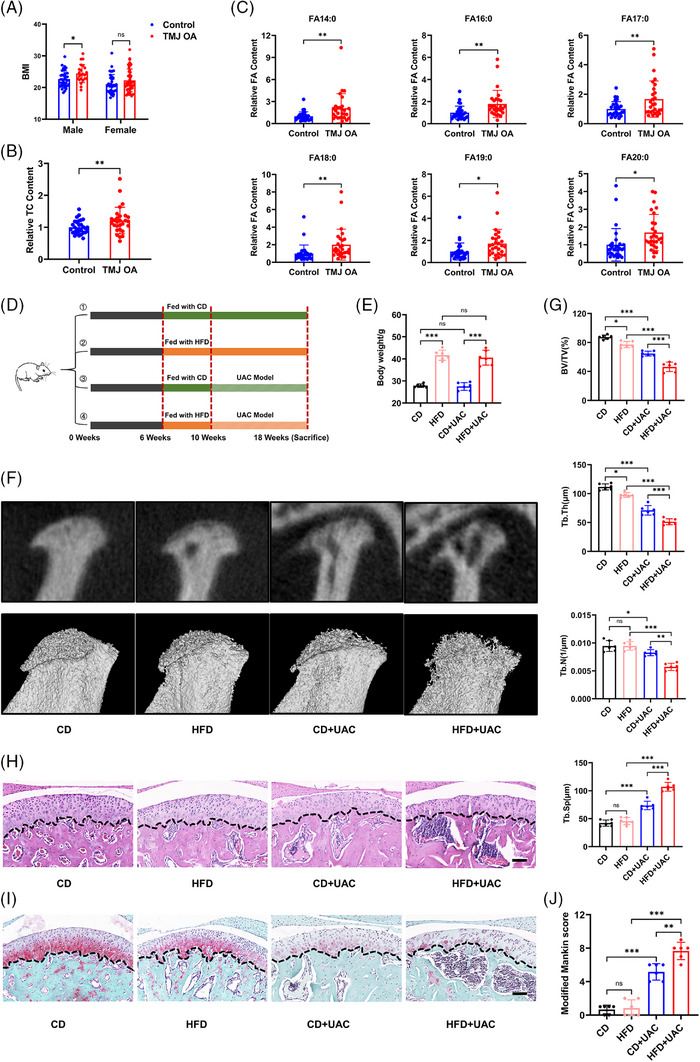
Obesity exacerbates the manifestations of TMJ OA. (A) Comparison of BMI between TMJ OA patients and healthy controls by gender (male patients, *n* = 23; male control group, *n* = 37; female patients, *n* = 41; female control group, *n* = 38). (B and C) Analysis of the plasma total cholesterol (B) and representative SFA (C) in TMJ OA patients compared (*n* = 28) to healthy controls (*n* = 29). (D) Animal experimental scheme. (E) Changes in body weight of mice in each group during the experimental period (*n* = 6). (F) Representative reconstruction images of the Micro‐CT analysis of trabeculae of condylar subchondral bone. (G) The value of BV/TV, Tb.Th, Tb.N and Tb.Sp of condylar subchondral bone (*n* = 6). (H and I) Representative H&E staining images (H) and SO staining images (I) of the sagittal plane of the condyle. Scale bar, 100 μm. (J) The modified Mankin score of the cartilage (*n* = 6). *p < .05; **p < .01; ***p < .001; ns, *p* > .05. Two‐way ANOVA followed by Tukey's multiple comparisons test (A, E, G, J), two‐tailed Student's unpaired *t*‐test analysis (B, C). Data are represented as mean ± SEM.

To verify the critical role of adipose tissue in obesity‐mediated aggravation of TMJ OA, we measured the body weight and height of TMJ OA patients and healthy controls from Nanjing Stomatological Hospital and compared their BMI values (body mass index, weight (kg)/height(m)^2^) (Table S3). As expected, TMJ OA patients had higher mean BMI values than healthy controls (Figure 1A), and this difference was significant in the male group (*p *< .05). Furthermore, we collected plasma samples from TMJ OA patients and healthy controls and then measured the levels of lipid metabolites in the plasma using HPLC–MS. Plasma metabolite analysis showed a noticeable increase in total cholesterol levels in individuals with TMJ OA compared with those in a healthy condition (Figure 1B). In comparing plasma FFAs between the two groups, numerous saturated fatty acids (SFAs), including palmitic and stearic acids, were markedly elevated in the plasma of TMJOA patients over healthy controls (Figures 1C and S3A). However, the levels of most types of unsaturated fatty acids were decreased in the plasma of TMJ OA patients, although this decrease was not statistically significant (Figure S3B,C). SFAs have a crucial function in the development of obesity and pose harm to the balance of metabolic processes.^42^ In fact, a clear correlation is noted between high SFA levels and human obesity, hepatic steatosis and type 2 diabetes.^43^ These data suggest that TMJ OA patients exhibit abnormal adipose metabolism and that their obesity‐related metabolic processes are significantly increased compared with those of healthy individuals.^44^, ^45^

To further confirm that obesity contributes to the development of TMJ OA, we established a TMJ OA mouse model using UAC and an obesity model using HFD as previously described^26^, ^27^ (Figure 1D). Following a 12‐week period of feeding HFD, the mice exhibited obvious obesity. UAC model did not influence the feeding or body weight of the mice, irrespective of whether they were on CD or HFD (Figure 1E).

Micro‐CT examination revealed that 12 weeks of HFD feeding slightly reduced the BV/TV of subchondral bone in the absence of UAC. After 8 weeks of UAC treatment in CD mice, bone resorption apparently occurred in the subchondral bone of the TMJ. Notably, HFD‐induced obesity significantly aggravated subchondral trabecular bone resorption and bone volume reduction during treatment with UAC (Figure 1F,G).

In the cartilage of the TMJ condyle, the UAC model successfully resulted in a rough cartilage surface, disordered chondrocyte arrangement and the loss of cartilage matrix. Obesity induced by HFD alone did not trigger significant pathological changes. However, in the presence of UAC, obesity considerably escalated the severity of cartilage impairment (Figure 1H,I). The modified Mankin's score revealed that HFD significantly aggravated UAC‐induced pathological changes in the TMJ (Figure 1J). Taken together, these data suggest that obesity accelerates TMJ OA development. However, after obesity exacerbates joint damage, weight loss achieved by changing diet did not reverse the pathological manifestations of TMJ OA (Figure S4).

### 3.2 Ad‐EVs from obese mice exacerbate the manifestations of TMJ OA

**FIGURE 2:**
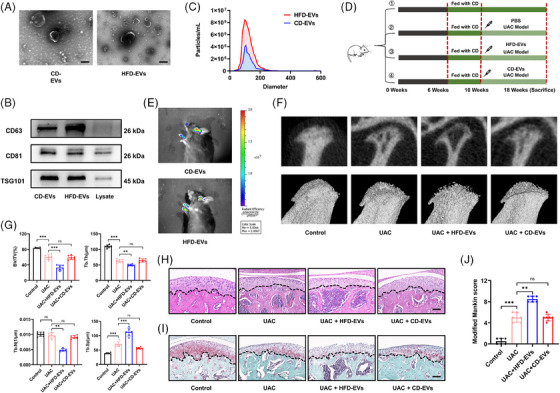
Ad‐EVs from obese mice exacerbate the manifestations of TMJ OA. (A) TEM analysis of CD‐EVs and HFD‐EVs. Scale bar, 100 nm. (B) The EVs‐related protein markers CD63, CD81 and TSG101 were measured by western blotting. (C) Particle size and distribution of CD‐EVs and HFD‐EVs analysed using nanoparticle tracking analysis. (D) Animal experimental scheme. (E) Fluorescence imaging of DiR‐labelled CD‐EVs and HFD‐EVs in TMJ area after 24 h of intravenous injection. (F) Representative reconstruction images of the Micro‐CT analysis of trabeculae of condylar subchondral bone. (G) The value of BV/TV, Tb.Th, Tb.N and Tb.Sp of condylar subchondral bone (*n* = 6). (H and I) Representative H&E staining images (H) and SO staining images (I) of the sagittal plane of the condyle. Scale bar, 100 μm. (J) The modified Mankin score of the cartilage (*n* = 6). *p < .05; **p < .01; ***p < .001; ns, *p* > .05. One‐way ANOVA followed by Tukey's multiple comparisons test (G, J). Data are represented as mean ± SEM.

It has been reported that adipose tissue is a critical regulator of osteoarthritis in the knee joint and that obese adipose tissue regulates pathological processes in other parts of the body by releasing EVs into the circulation.^46^, ^47^ To determine whether Ad‐EVs exacerbate pathological changes in TMJ OA, we collected EVs from the epididymal adipose tissue of both lean mice (CD‐EVs) and obese mice (HFD‐EVs). CD‐EVs and HFD‐EVs were approximately 50−200 nm in diameter, and both expressed the EV marker proteins CD63, CD81 and TSG101 (Figure 2A,B). Notably, the concentration of HFD‐EVs derived from per unit mass of adipose tissue was much higher than that of CD‐EVs (Figure 2C).

Next, we injected 100 μL of CD‐EVs or HFD‐EVs (1 μg/μL) into UAC mice via the tail vein once per week^40^, ^48^ (Figure 2D). In vivo fluorescence imaging and confocal microscopy revealed that both CD‐EVs and HFD‐EVs were successfully distributed to the TMJ area 24 h after injection (Figures 2E and S5). Consistent with the effect of HFD‐induced obesity, HFD‐EVs injection had a negative effect on subchondral bone remodelling of the TMJ condyle. Compared with UAC alone or UAC+CD‐EVs, infusion of HFD‐EVs noticeably decreased the fraction of bone volume, and the quantity and thickness of trabeculae, while it significantly escalated the separation of the trabeculae (Figure 2F,G). In contrast, CD‐EVs had no significant effect on subchondral bone remodelling. HFD‐EVs aggravated condylar cartilage damage. Compared with those in the UAC group, the cartilage surface layer in the UAC+HFD‐EVs group was more heterogeneous, a reduced number of chondrocytes was observed in the cartilage layer, and the extracellular matrix was also significantly degraded (Figure 2H,I). The modified Mankin's score revealed that the pathological grade of the TMJ in UAC+HFD‐EVs group was notably elevated compared with UAC group and UAC+CD‐EVs group (Figure 2J). These results suggest that HFD‐EVs play a similar role in the development of osteoarthritis as that of HFD‐induced obesity. Adipose tissue in the obese state may aggravate the development of osteoarthritis by releasing EVs into the circulation.

### 3.3 Ad‐EVs from obese mice affect chondrocyte metabolism and induce chondrocyte apoptosis

The homeostasis of the TMJ is inseparable from chondrocytes viability and their ability to balance the synthesis and degradation of the cartilage matrix.^49^ To explore whether Ad‐EVs regulate chondrocyte viability, we collected CD‐EVs and HFD‐EVs and used them to treat ATDC5 chondrocytes.

**FIGURE 3:**
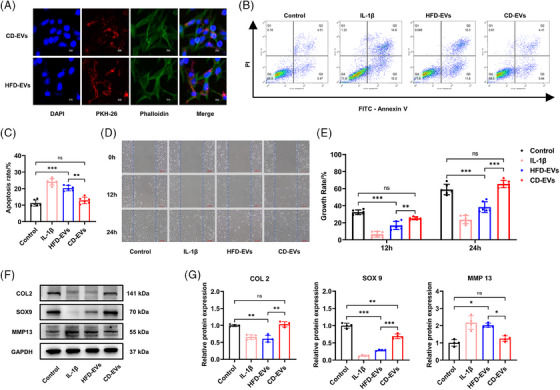
Ad‐EVs from obese mice affect chondrocyte metabolism and lead to chondrocyte apoptosis. (A) Representative confocal images of chondrocytes incubated with PKH26‐labelled CD‐EVs and HFD‐EVs for 24 h. Chondrocytes were fixed and stained with phalloidin for actin (green) and DAPI (blue) staining for nuclei. Scale bar, 10 μm. (B and C) The effect of CD‐EVs and HFD‐EVs on chondrocyte apoptosis was detected by flow cytometry and statistically analysed (*n* = 6). (D and E) The representative images showing the proliferation and migration ability of chondrocytes were obtained by the scratch test and statistically analysed (*n* = 6). Scale bar, 100 μm. (F and G) The protein levels of COL2, SOX9 and MMP13 in chondrocyte was detected by WB assay and statistically analysed (*n* = 4). *p < .05; **p < .01; ***p < .001; ns, *p* > .05. One‐way ANOVA followed by Tukey's multiple comparisons test (G, J). Data are represented as mean ± SEM.

CD‐EVs and HFD‐EVs labelled with PKH‐26 were taken up by chondrocytes within 12 h (Figure 3A). Flow cytometry revealed that HFD‐EVs exerted similar effects as IL‐1β, which significantly increased the proportion of apoptotic chondrocytes (*p *< .05). Although CD‐EVs slightly increased the rate of chondrocyte apoptosis, the change was not of statistical significance (*p *> .05; Figure 3B,C). The influence of Ad‐EVs on chondrocyte proliferation and migration were analysed using a scratch assay, as previously described. We found that HFD‐EVs, although less severe than IL‐1β, significantly inhibited chondrocyte proliferation and migration at 12 and 24 h (*p *< .05). In consistent, CD‐EVs had no apparent effect on chondrocytes in these contexts (*p *> .05; Figure 3D,E). We next examined the levels of COL2, SOX9 and MMP13 in chondrocytes, which are key molecules associated with cartilage homeostasis.^50^, ^51^, ^52^ HFD‐EVs inhibited COL2 and SOX9 expression but up‐regulated MMP13 levels. CD‐EVs reduced SOX9 expression levels, but this reduction was significantly less than that induced by HFD‐EVs. In addition, CD‐EVs did not significantly affect COL2 and MMP13 expression in chondrocytes (Figure 3F,G). We also investigated the effects of the EVs from CD and HFD mice on osteogenic function of osteoblasts and bone mesenchymal stem cells (BMSCs). The results (Figure S6) revealed that EVs from obese adipose tissue exhibited significant inhibitory effects on osteogenesis in osteoblasts and BMSCs, while CD‐EVs had a slight promoting effect on osteogenesis. These results are consistent with previous studies.^18^

Adipocyte is a key cell type in adipose tissue and constitute the main source of EVs‐derived miRNAs in the circulation.^53^, ^54^ We isolated adipose tissue from CD and HFD mice, minced the tissue and co‐cultured it with chondrocytes. Pretreatment with GW4869, a sphingomyelinase inhibitor that effectively blocks EVs release, was performed 24 h earlier to inhibit the secretion of EVs from CD‐adipocytes and HFD‐adipocytes^55^ (Figure S7A). Co‐culture with HFD‐adipocytes increased the proportion of apoptotic chondrocytes, and GW4869 partially counteracted this effect (Figure S7B,C). CD‐adipocytes had no significant effect on chondrocyte apoptosis rates regardless of the presence of GW4869 (Figure S7B,C). Scratch assay results showed that GW4869 counteracted the restrictive impact of HFD‐adipocytes on chondrocyte proliferation and migration at 12 and 24 h. Blocking EVs secretion in CD‐adipocytes limited the growth of co‐cultured chondrocytes during the first 12 h, but the limitation was not significant after 24 h (Figure S7D,E). In terms of protein changes in chondrocytes, co‐culture with HFD‐adipocytes yielded effects similar to those of HFD‐EVs treatment, which decreased COL2 and SOX9 expression and increased MMP13 expression. These changes were reversed by inhibiting the secretion of EVs from HFD‐adipocytes (Figure S7F,G). Co‐culture with CD‐adipocytes alone did not have a noteworthy influence on the aforementioned proteins(*p *> .05). Interestingly, inhibiting EVs secretion from CD‐adipocytes decreased COL2 and SOX9 expression and increased MMP13 expression in chondrocytes (Figure S7F,G). Our findings corroborated that Ad‐EVs were involved in the interaction between adipose tissue and chondrocytes.

### 3.4 Conditional knockout of *Ggpps* reduces Ad‐EVs in obese mice and attenuates obesity‐promoted TMJ OA

**FIGURE 4:**
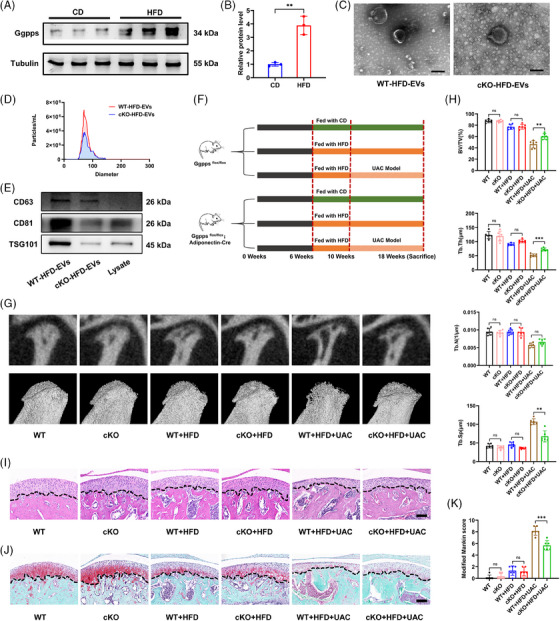
Conditional knockout of *Ggpps* reduces Ad‐EVs in obese mice and attenuates obesity‐promoted TMJ OA. (A and B) The protein levels of *Ggpps* in adipose tissue was detected by WB assay and statistically analysed (*n* = 3). (C) TEM analysis of HFD‐EVs from WT and cKO mice. Scale bar, 100 nm. (D) The EVs‐related protein markers CD63, CD81 and TSG101 were measured by western blotting. (E) Particle size and distribution of HFD‐EVs from WT and cKO mice analysed using nanoparticle tracking analysis. (F) Animal experimental scheme. (G) Representative reconstruction images of the Micro‐CT analysis of trabeculae of condylar subchondral bone. (H) The value of BV/TV, Tb.Th, Tb.N and Tb.Sp of condylar subchondral bone (*n* = 5–7). (H and I) Representative H&E staining images (I) and SO staining images (J) of the sagittal plane of the condyle. Scale bar, 100 μm. (K) The modified Mankin score of the cartilage (*n* = 5–7). *p < .05; **p < .01; ***p < .001; ns, *p* > .05. Two‐tailed Student's unpaired *t*‐test analysis (B). Two‐way ANOVA followed by Tukey's multiple comparisons test (H, K). Data are represented as mean ± SEM.

We previously reported that *Ggpps* is essential for the release of EVs and can affect metabolic processes in other organs.^26^ Here, we revealed that *Ggpps* expression in the adipose tissue of obese mice exceeded that of healthy mice (Figure 4A,B). To evaluate the physiological effect of adipose tissue‐derived EVs on TMJ tissue, we established adipocyte‐specific *Ggpps*‐knockout (cKO) mice to investigate whether altering the secretion of HFD‐EVs in vivo affects the pathological state of TMJ OA. After a 12‐week period of feeding HFD, the weight of body and adipose tissue of cKO mice were markedly less than those of the WT mice (Figure S8A,B). Knockdown of *Ggpps* had no significant effect on the morphology of adipocytes and knee cartilage in both CD and HFD conditions (Figure S8C,D).

As expected, *Ggpps* knockout resulted in a marked reduction in the amount of Ad‐EVs in obese mice (Figure 4C–E). To verify the effect of EVs depletion on TMJ OA, we fed WT and cKO mice with an HFD and constructed UAC models (Figure 4F). No difference in condylar subchondral bone was noted between WT and cKO mice in the CD group. When fed an HFD, cKO mice presented greater trabecular thickness and smaller trabecular separation; however, there was no noticeable difference in the total bone volume between the two groups (*p *> .05). When mechanical stimulation of UAC was performed in combination with HFD, WT mice lost subchondral bone as previously described. Although the cKO‐HFD‐UAC mice, presented subchondral bone loss, they presented a significantly increased BV/TV and Tb.Th and a decreased Tb.Sp than WT‐HFD‐UAC mice (Figure 4G,H). In the cartilage layer, no significant difference was noted between cKO mice and WT mice under CD or HFD conditions. In response to HFD+UAC stimulation, the cKO mice presented a thicker cartilage layer with more extracellular matrix deposition than WT mice (Figure 4I,J). The modified Mankin's score revealed that *Ggpps* knockout in adipose tissue significantly reversed the pathological changes caused by HFD+UAC (Figure 4K). These data suggest that *Ggpps*‐mediated Ad‐EVs release is involved in obesity‐mediated promotion of osteoarthritis.

### 3.5 Inhibition of miR‐3074‐5p prevents pathological damage of TMJ OA promoted by HFD‐EVs

**FIGURE 5:**
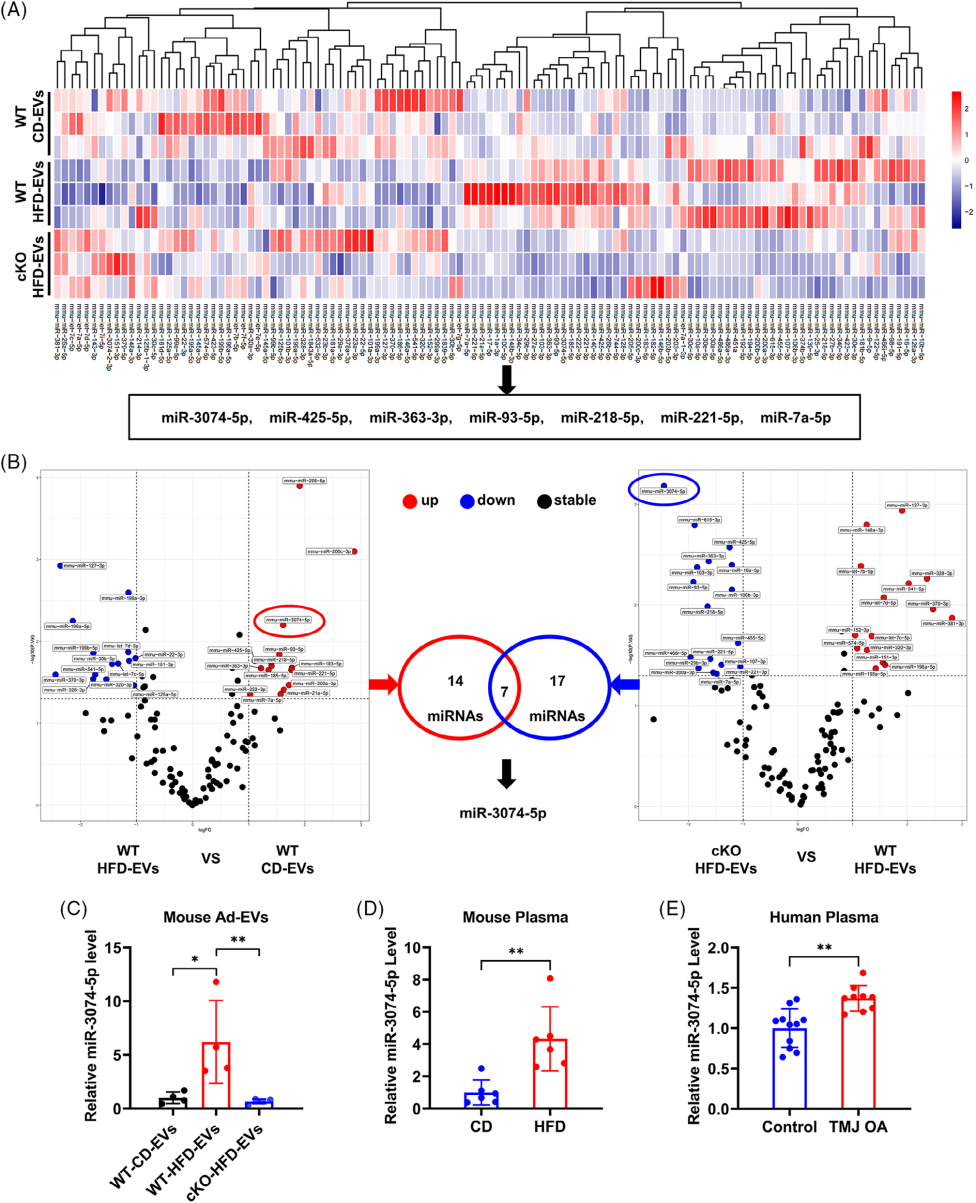
Identification of miRNAs in Ad‐EVs that play a role in obesity‐related TMJ OA. (A) Cluster heat map of differentially expressed miRNAs in CD‐EVs and HFD‐EVs from WT mice and HFD‐EVs from cKO mice (*n* = 3). (B) Volcano plots were used to show the miRNAs in Ad‐EVs that were up‐regulated in the obese state and decreased in response to specific knockdown of *Ggpps* in adipocytes (*n* = 3). (C) miR‐3074‐5p level in the Ad‐EVs of mice (*n* = 4). (D) miR‐3074‐5p level in the plasma from HFD and CD mice. (E) miR‐3074‐5p level in the plasma from TMJ OA patients (*n* = 9) and healthy controls (*n* = 11). *p < .05; **p < .01; ***p < .001; ns, *p* > .05. One‐way ANOVA followed by Tukey's multiple comparisons test (C), two‐tailed Student's unpaired *t*‐test analysis (D, E). Data are represented as mean ± SEM.

The regulatory effect of Ad‐EVs on receptor organisation mainly depends on their cargo miRNAs.^53^ To explore the intrinsic mechanism by which HFD‐EVs promote the development of TMJ OA, we performed high‐throughput miRNA sequencing to predict potential miRNAs that were altered in Ad‐EVs. The sequencing results indicated that a sum of 117 miRNAs displayed varied expression across the three groups of Ad‐EVs (Figure 5A). Further analysis (Figure 5B) revealed that seven EVs‐miRNAs (miR‐3074‐5p, miR‐425‐5p, miR‐363‐3p, miR‐93‐5p, miR‐218‐5p, miR‐221‐5p, miR‐7a‐5p) were up‐regulated in the obese state but were down‐regulated after knocked out of the *Ggpps* gene in adipocytes. Given that the change in miR‐3074‐5p was the most significant in the two differential analyses (with the smallest overall *p* value) and that miR‐3074‐5p in rheumatoid arthritis patients was up‐regulated compared with healthy controls,^56^ we premised that miR‐3074‐5p exerts a controlling influence in TMJ OA. The increase of miR‐3074‐5p in HFD‐EVs was verified using qPCR (Figure 5C) and miR‐3074‐5p quantities in obese mice plasma were noticeably higher compared with those in lean mice (Figure 5D). Importantly, patients with TMJ OA presented elevated plasma miR‐3074‐5p levels compared with healthy individuals (Figure 5E).

**FIGURE 6:**
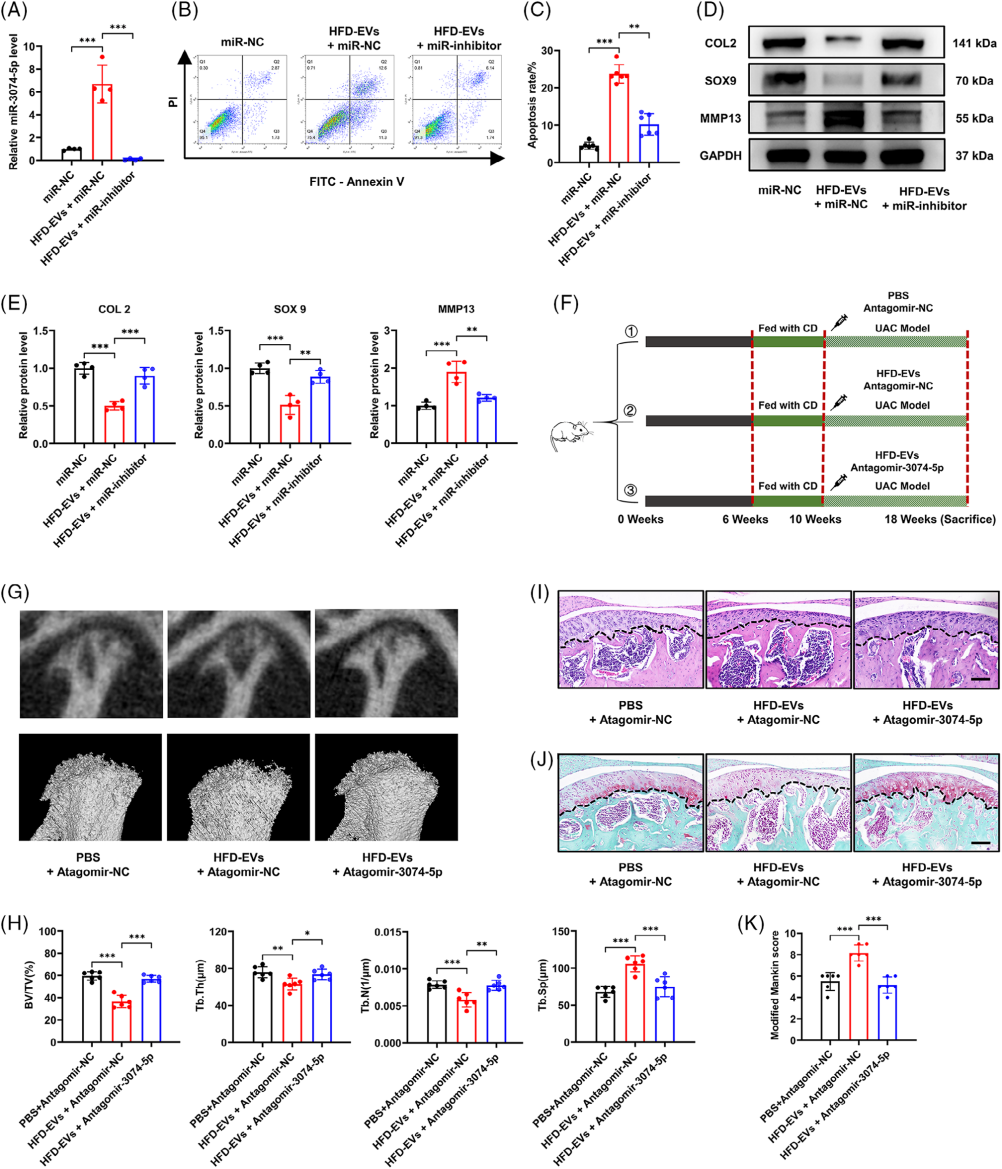
Inhibition of miR‐3074‐5p prevents pathological damage of TMJ OA promoted by HFD‐EVs. (A) Chondrocytes were treated with or without HFD‐EVs and transfected with miR‐inhibitor or miR‐NC of miR‐3074‐5p for 24 h. The miRNA level was determined by qRT‐PCR (*n* = 4). (B and C) The effect of miR‐3074‐5p on chondrocyte apoptosis was detected by flow cytometry and statistically analysed (*n* = 6). (D and E) The protein levels of COL2, SOX9 and MMP13 in chondrocyte was detected by WB assay and statistically analysed (*n* = 4). (F) Animal experimental scheme. (G) Representative reconstruction images of the Micro‐CT analysis of trabeculae of condylar subchondral bone. (H) The value of BV/TV, Tb.Th, Tb.N and Tb.Sp of condylar subchondral bone (*n* = 6). (H and I) Representative H&E staining images (I) and SO staining images (J) of the sagittal plane of the condyle. Scale bar, 100 μm. (K) The modified Mankin score of the cartilage (*n* = 6). *p < .05; **p < .01; ***p < .001; ns, *p* > .05. One‐way ANOVA followed by Tukey's multiple comparisons test (A, C, E, H, K). Data are represented as mean ± SEM.

We subsequently constructed a specific miRNA inhibitor to investigate the influence of miR‐3074‐5p on chondrocytes. The qPCR results revealed that treatment with HFD‐EVs for 24 h substantially elevated the levels of miR‐3074‐5p in chondrocytes, which was successfully reversed by the miR‐3074‐5p inhibitor (Figure 6A). Flow cytometric analysis demonstrated that miR‐3074‐5p inhibitor diminished HFD‐EVs‐triggered apoptosis in chondrocytes (Figure 6B,C). In addition, administration of the miR‐3074‐5p inhibitor increased COL2 and SOX9 expression and reduced the levels of catabolic proteins such as MMP13 (Figure 6D,E).

We conducted further research to determine if miR‐3074‐5p participated in HFD‐EVs‐mediated TMJ OA by injecting antagomir‐3074‐5p in a mouse model. Antagomir‐NC and antagomir‐3074‐5p (10 μL at a concentration of 4 mM) were injected into the TMJs of mice with mechanically induced TMJ OA alone or in combination with intravenous injection of PBS or HFD‐EVs as indicated^57^, ^58^ (Figure 6F). H&E staining results showed that the antagomiRNAs had no significant side effects on the morphology of the liver, spleen or kidney tissues of the mice (Figure S9). Micro‐CT revealed that intra‐articular injection of antagomir‐3074‐5p markedly reduced the effect of HFD‐EVs‐mediated subchondral bone resorption in UAC‐induced TMJ OA mice (Figure 6G,H). Moreover, our results demonstrated that the HFD‐EVs‐mediated decrease in cartilage matrix and increase in pathological severity were ameliorated by intra‐articular injection of antagomir‐3074‐5p (Figure 6I–K). These evidences indicate that the exacerbation of TMJ degeneration triggered by HFD‐EVs is highly dependent on miR‐3074‐5p.

### 3.6 MiR‐3074‐5p encapsulated in the HFD‐EVs aggravate TMJ OA by inhibiting SMAD4 expression

**FIGURE 7:**
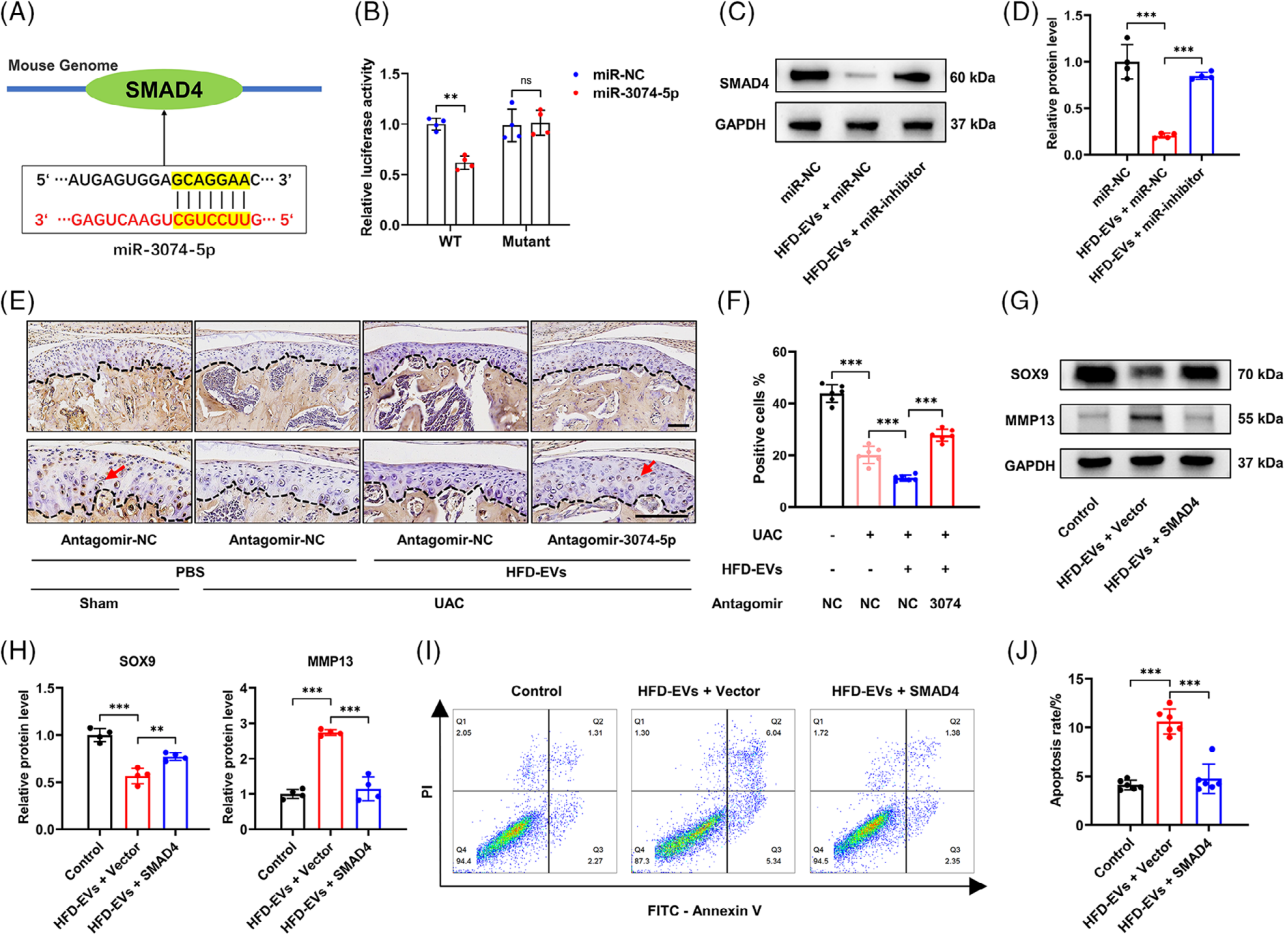
MiR‐3074‐5p encapsulated in the HFD‐EVs aggravates TMJ OA by inhibiting SMAD4 expression. (A) Schematic representation of the predicted binding site of miR‐3074‐5p in the 3′UTR of SMAD4 gene. (B) Chondrocytes were transfected with WT or Mut 3′‐UTR of SMAD4 and miR‐3074‐5p or miR‐NC, luciferase activity was detected by Dual‐Luciferase Reporter Assay System (*n* = 4). (C and D) Chondrocytes were treated with HFD‐EVs and transfected with miR‐inhibitor of miR‐3074‐5p for 24 h, then the relative expression of SMAD4 was detected by WB assay (*n* = 4). (E and F) The representative images of IHC staining and quantification of SMAD4 in chondrocytes of TMJ cartilage sections. Red arrowheads indicate the positive cells. Scale bar, 100 μm. (G and H) The protein levels of SOX9 and MMP13 in chondrocytes was detected by WB assay and statistically analysed (*n* = 4). (I and J) The effect of SMAD4 overexpression on chondrocyte apoptosis was detected by flow cytometry and statistically analysed (*n* = 6). *p < .05; **p < .01; ***p < .001; ns, *p* > .05. Two‐way ANOVA followed by Tukey's multiple comparisons test (B). One‐way ANOVA followed by Tukey's multiple comparisons test (D, F, H, J). Data are represented as mean ± SEM.

Drosophila mothers against decapentaplegic homolog 4 (SMAD4) is an intracellular effector of TGF‐β family. The absence of SMAD4 triggers cell apoptosis and escalates the progression of osteoarthritis.^59^ According to the database of miRNA targets, miR‐3074‐5p might target SMAD4 during the course of osteoarthritis (Figure 7A). We constructed a luciferase reporter plasmid to confirm that the 3ʹ‐UTR of SMAD4 was the direct target of miR‐3074‐5p. A luciferase reporter plasmid was assembled to verify that the SMAD4 gene is directly targeted by miR‐3074‐5p. The findings demonstrated that the luciferase activity of the WT SMAD4 plasmid was significantly decreased upon co‐transfection with miR‐3074‐5p. Conversely, the MUT SMAD4 plasmid remained unaffected by miR‐3074‐5p (Figure 7B). WB results showed that HFD‐EVs treatment reduced SMAD4 gene expression in chondrocytes. Interestingly, transfection of the miR‐3074‐5p inhibitor counterbalanced the impact of HFD‐EVs on SMAD4 (Figure 7C and D). Next, we investigated whether inhibiting miR‐3074‐5p exerted similar effects on SMAD4 in vivo. Immunohistochemical examination demonstrated a notable decrease in the percentage of SMAD4‐positive chondrocytes in UAC mice compared with the control mice. In addition, the HFD‐EVs induced reduction of SMAD4 expression in TMJ OA mice was reversed by intra‐articular injection of antagomir‐3074‐5p (Figure 7E,F).

**FIGURE 8:**
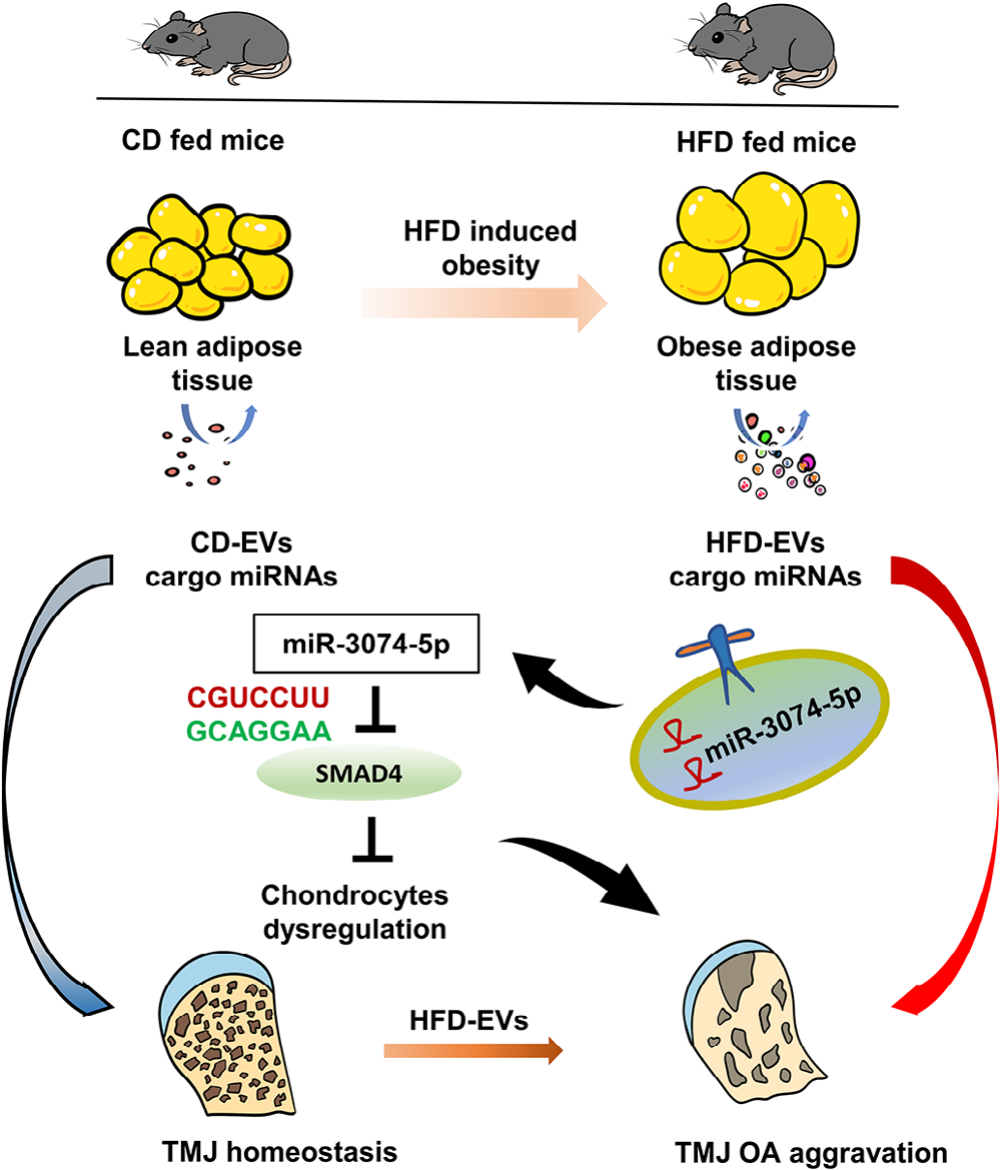
Schematic illustration for the mechanism of adipose tissue derived exosomal miR‐3074‐5p promoting TMJ OA aggravation.

To address whether the increased degeneration in miR‐3074‐5p enriched chondrocytes is dependent on SMAD4 inhibition, we generated an overexpression plasmid targeting SMAD4 and a blank vector. Compared with blank vector, overexpression of SMAD4 increased SOX9 protein levels and decreased MMP13 protein levels when co‐treatment with HFD‐EVs in chondrocytes (Figure 7G,H). Moreover, flow cytometry revealed that SMAD4 overexpression reduced the degree of chondrocyte apoptosis induced by HFD‐EVs (Figure 7I,J). Taken together, these findings implied that miR‐3074‐5p in HFD‐EVs could aggravate TMJ OA progression by targeting the SMAD4 pathway in chondrocytes (Figure 8).

## 4 DISCUSSION

At present, TMJ OA treatment involves alleviating pain and restoring joint function. Owing to a limited understanding of its pathogenesis, there is no effective pharmacological strategy to attenuate the pathological changes in TMJ OA.^60^ Accumulating evidence has demonstrated that obesity and metabolic factors play important roles in TMJ disorders, but the underlying mechanisms are poorly understood.^12^, ^14^, ^15^ Adipose tissue secretes various bioactive factors that mediate crosstalk between adipose tissue and other organs, including bone and cartilage.^61^, ^62^, ^63^ This study first demonstrated that adipose tissue dysfunction plays an important role in the pathogenesis of TMJ OA.

Our research indicated an increased prevalence of overweight among patients with TMJ OA compared with healthy individuals, with male TMJ OA patients displaying a higher average BMI than their healthy male counterparts. Moreover, our subsequent experiments have proved that obesity is an important promoting factor in TMJ OA pathology. We also noticed that a proportion of TMJ OA patients did not have an obese BMI value and that obesity induced by HFD alone in mice did not trigger significant cartilage damage. This suggests that obesity alone is not an independent driver for TMJ OA. When the joint homeostasis is disrupted, obese adipose tissue could exacerbate the severity of TMJ OA. The BMI difference between TMJ OA patients and healthy controls was not evident in the female group. This observation could be associated with gender differences in oestrogen‐dependent regulation of TMJ OA. High oestrogen levels in women may be one of the causes of TMJ OA.^64^ Given the significant regulatory role of oestrogen in TMJ OA,^65^, ^66^ we exclusively used male mice in our animal experiments. After obesity exacerbates joint damage, weight loss by changing diets does not reverse the pathological manifestations of TMJ OA. This result may be related to the special hypovascular structure of the joint. As a degenerative disease, osteoarthritis is extremely difficult to reverse once pathological degeneration occurs.^67^, ^68^

By coculturing chondrocytes with adipocytes, we demonstrated that adipocytes from HFD‐fed mice disrupted the metabolic activity of chondrocytes and induced chondrocyte apoptosis. The metabolic homeostasis of chondrocytes is fundamental for joint health. Enhancing the knowledge of the molecular processes through which adipose tissue participates in chondrocyte injury is essential for the development of innovative strategies to mitigate the pathological damage associated with TMJ OA. Recently, adipose tissue has been reported to actively secrete EVs and serves as the main source of circulating EVs.^40^, ^53^ In obese rodents and obese humans, Ad‐EVs are associated with the progression of multiple diseases. Wang et al.^19^ reported that Ad‐EVs from obese mice or diabetes patients with can be transferred to the brain and are enriched in neurons, inducing marked synaptic loss and cognitive dysfunction in obesity‐related insulin resistance. However, few studies have been conducted to delineate whether obese Ad‐EVs mediate interorgan crosstalk with joints, particularly non‐weight‐bearing joints such as the TMJ. In this research, it was noted that Ad‐EVs could be transferred to the TMJ region via the circulatory system. Interestingly, HFD‐EVs accelerated cartilage destruction and subchondral bone remodelling during TMJ OA. By in vitro experiments, we found that HFD‐EVs not only promoted chondrocyte apoptosis but also increased catabolic activity and decreased the anabolic activity of chondrocytes. In previous reports, adipose tissue was implicated in the onset and progression of osteoarthritis through the release of adipokines and inflammatory cytokines.^47^ Here, we revealed a novel regulatory role of adipose tissue in which HFD‐EVs aggravated mechanical stress‐induced TMJ OA without causing inflammation.

The formation and release of EVs involve the transport, fusion and interaction of endosome vesicles.^69^ *Ggpps* is an important protein‐modifying enzyme that regulates the fusion of intracellular vesicles to the cell membrane through the downstream protein Rab27A.^70^, ^71^ *Ggpps* expression is markedly elevated in the adipose tissue of obese mice.^72^ We recently showed that *Ggpps* knockdown inhibited the secretion of hepatic EVs and thereby reduced lipid deposition in adipocytes.^26^ In the present study, we suggest that Ad‐EVs mediated by *Ggpps* are novel mediators of obesity‐accelerated TMJ OA. In the absence of mechanical destruction, *Ggpps* knockdown in HFD mice had no effect on the cartilage morphology of the knee joint, which was similar to the results of TMJ. This may be due to the homeostatic regulation of chondrocyte and extracellular matrix damage.^73^ This means that under the milder range of adverse conditions, cartilage could maintain homeostasis without damage.

Adipocytes constitute the main cell type in adipose tissue that performs secretory functions.^74^ In previous studies, to inhibit the secretion of Ad‐EVs in vivo, GW4869 was commonly administered by intraperitoneal injection.^19^, ^75^ However, in vivo administration of GW4869 may nonspecifically inhibit EVs secretion in multiple organs.^19^, ^75^, ^76^ For example, intravenous injection of GW4869 inhibits the release of EVs from neurons in the brain, thereby specifically affecting the function of osteoprogenitors in bone healing.^77^ Gan et al.^78^ showed the intraperitoneal injection of GW4869 can be used to inhibit the release of EVs from cardiomyocytes into the circulation. To specifically investigate the function of Ad‐EVs in TMJ OA, we used the Flox–Cre system to knock out the *Ggpps* gene in adipocytes. As expected, *Ggpps* knockout in adipocytes significantly inhibited the secretion of Ad‐EVs in HFD fed mice. By constructing UAC and obese models in WT and cKO mice, we found that the inhibition of adipose tissue‐derived EVs secretion significantly reversed the HFD‐mediated promotion of TMJ OA. These findings suggest that EVs is a key factor in adipose tissue that mediate the pathological process of obesity‐accelerated TMJ OA. Therefore, it is conceivable that altering Ad‐EVs might serve as a joint‐targeted therapy of TMJ OA.

Studies have indicated that Ad‐EVs serve as the principal source of circulating miRNAs in both humans and mice.^53^, ^79^ The types and numbers of miRNAs in Ad‐EVs between obese and normal individuals are not consistent. Wei et al.^40^ reported that EVs derived from obese mice contained more proinflammatory miRNAs than those derived from lean mice, and the increase in proinflammatory miRNAs promoted the severity of colitis in these mice. Our miRNA sequencing showed that the miRNA expression profile of HFD‐EVs was altered, confirming that adipose tissue can secrete EVs with different functions under obesity conditions. Subsequent studies indicated that miR‐3074‐5p levels were elevated in HFD‐EVs and that *Ggpps* gene knockout in adipocytes reversed this increasing trend. Inhibiting miR‐3074‐5p in chondrocytes reversed the metabolic damage induced by HFD‐EVs and inhibited chondrocyte apoptosis. Notably, previous studies have shown that miR‐3074‐5p is associated with iron overload‐induced osteoblast apoptosis.^80^ Jiang et al.^56^ reported that miR‐3074‐5p expression in rheumatoid arthritis patients was up‐regulated compared with that in healthy controls. These findings are consistent with our results, which suggest that miR‐3074‐5p is likely involved in chondrocyte apoptosis and inhibiting miR‐3074‐5p prevented the exacerbation of osteoarthritis‐like damage in HFD‐fed mice. Thus, we suggest that the effects of HFD‐EVs on chondrocytes and the TMJ are mediated through miR‐3074‐5p.

In general, miRNAs in Ad‐EVs play a regulatory role by interacting with target genes in cells. Based on results from a target gene prediction website as well as in vitro and in vivo experiments, we confirmed that SMAD4 is a target gene of miR‐3074‐5p. SMAD4 is closely related to bone and cartilage development.^59^, ^81^ SMAD4 affects chondrocyte morphology and enhances chondrocyte migration.^82^ More critically, SMAD4 expression has been reported to be down‐regulated in the articular cartilage of osteoarthritis patients,^83^ and TGF‐β/SMAD4 pathway activation alleviated the progression of diabetes‐related osteoarthritis.^84^ Moreover, SMAD4 overexpression inhibited LPS‐induced chondrocyte apoptosis in vitro.^85^ Therefore, SMAD4 is a key factor in chondrocyte apoptosis and osteoarthritis development. These findings suggest that SMAD4 is a critical target that may be involved in miR‐3074‐5p‐mediated TMJ lesions.

## 5 CONCLUSION

This study demonstrated a previously unknown mechanism by which obese adipose tissue exacerbates the pathogenesis of TMJ OA (Figure 8). During obesity, the quantity and function of *Ggpps*‐mediated Ad‐EVs are altered. The increased levels of miR‐3074‐5p carried by HFD‐EVs enters the TMJ region and accelerates cartilage destruction by targeting SMAD4. Targeting Ad‐EVs and their specific miRNAs may represent a promising therapeutic strategy for obesity‐related TMJ OA. There are still limitations to this study. Adipose tissue is made up of several cell types such as adipocytes, endothelial cells and immune cells. However, whether EVs derived from different cells play different roles has not been investigated in this study. We will continue to explore these problems in our future research.

## AUTHOR CONTRIBUTIONS

Baochao Li, Yue Zhao and Huang Li conceived and designed the studies. Baochao Li, Yuqin Jin, Bingqing Zhang, Jialing Li, Jingzi Zhang, Yanyi Wang and Yiwen Zhou performed experiments and data analysis. Baochao Li, Yuqin Jin and Bingqing Zhang wrote the manuscript. Tong Lu, Bingqing Zhang and Caixia Zhang analysed clinical samples and reviewed the data. Yue Zhao reviewed analysis results and revised the manuscript. Huang Li supervised the overall study and approved the manuscript. Baochao Li, Yuqin Jin and Bingqing Zhang were assigned co‐first authorship because their contributions were considered equally essential to this project. The order of the co‐first authors was determined by their efforts and duration of work on this project. All authors have reviewed and approved the manuscript.

## CONFLICT OF INTEREST STATEMENT

The authors have declared that no conflict of interest exists.

## ETHICS STATEMENT

This study protocol was approved by the Institute Research Ethics Committee of the Nanjing Stomatological Hospital (NJSH‐2022NL‐39). All animal studies and experimental protocols were approved and conducted according to the Animal Care and Use Committee of Nanjing University in accordance with institutional animal care and use committee guidelines (IACUC‐D2204005).

## Supporting information



Supporting Information

Supporting Information

Supporting Information

## Data Availability

Data are available upon reasonable request. The data that support the findings in this study are available from the corresponding author upon request.
