# Supplementary material for: Adipose tissue‐derived extracellular vesicles aggravate temporomandibular joint osteoarthritis associated with obesity
Source: Clin Transl Med. 2024 Sep 30;14(10):e70029. doi: 10.1002/ctm2.70029 (PMC11442491; doi:10.1002/ctm2.70029)
Supplement: Supplementary file 2 — Supporting Information [file CTM2-14-e70029-s001.docx]

Supplemental table 1

Antibody list.

| **Antibody** | **Source** | **Identifier** |
| --- | --- | --- |
| CD63 antibody | Abcam | ab134045 |
| CD81 antibody | Abcam | ab109201 |
| TSG101 antibody | Abcam | ab125011 |
| SOX9 antibody | Abcam | ab185966 |
| COL2 antibody | Proteintech | 28459-1-AP |
| MMP13 antibody | Proteintech | 18165-1-AP |
| GGPPS antibody | Santa Cruz | sc-271680 |
| SMAD4 antibody | Abcam | ab40759 |
| GAPDH antibody | Proteintech | 10494-1-AP |
| β-Tubulin | ABclonal | AC008 |
| m-IgGκ BP-HRP | Santa Cruz | sc-516102 |
| Anti-rabbit IgG-HRP antibody | Abcam | ab6721 |

Supplemental table 2

Primer list.

| **Primer** | **Source** | **Oligonucleotides** |
| --- | --- | --- |
| MiR-3074-5p Step-loop Primer | GenScript | GTCGTATCCAGTGCAGGGTCCGAGGTA  TTCGCACTGGATACGACACTGGC |
| U6 Step-loop Primer | GenScript | AACGCTTCACGAATTTGCGT |
| MiR-3074-5p Forward Primer | GenScript | GCGGTTCCTGCTGAACTGA |
| MiR-3074-5p Reverse Primer | GenScript | AGTGCAGGGTCCGAGGTATT |
| U6 Forward Primer | GenScript | CTCGCTTCGGCAGCACA |
| U6 Reverse Primer | GenScript | AACGCTTCACGAATTTGCGT |
| SMAD4 Forward Primer | GenScript | ACACCAACAAGTAACGATGCC |
| SMAD4 Reverse Primer | GenScript | GCAAAGGTTTCACTTTCCCCA |
| GAPDH Forward Primer | GenScript | TGTGTCCGTCGTGGATCTGA |
| GAPDH Reverse Primer | GenScript | TTGCTGTTGAAGTCGCAGGAG |
