# Supplementary material for: Adipose tissue‐derived extracellular vesicles aggravate temporomandibular joint osteoarthritis associated with obesity
Source: Clin Transl Med. 2024 Sep 30;14(10):e70029. doi: 10.1002/ctm2.70029 (PMC11442491; doi:10.1002/ctm2.70029)
Supplement: Supplementary file 1 — Supporting Information [file CTM2-14-e70029-s002.docx]

Supplementary figure 1. Selection of the region of interest (ROI) in micro-CT analysis. (A) Representative sagittal ROI image of the TMJ condyle. (B) Representative coronal ROI image of the TMJ condyle. (C) The ROI were acquired and measured in 3D images.

Supplemental figure 2. Detection of the chondrogenic capability of ATDC5 cells in vitro. (A). The representative images of Alcian blue staining of ATDC5 cells after 0 and 7 days of chondrogenic induction. Scale bar, 100 µm. (B). Chondrogenic mRNA expression of ATDC5 cells after 0 and 7 days of chondrogenic induction (n=3). * P<0.05; ** P<0.01; *** P<0.001; ns, P > 0.05. One-way ANOVA followed by Tukey’s multiple comparisons test. Data are represented as mean ± SEM.

Supplemental figure 3. Comparison of plasma FFA between TMJ OA patients and healthy controls. (A) Saturated fatty acids; (B) Monounsaturated fatty acids. (C) Polyunsaturated fatty acids. * P<0.05; ** P<0.01; *** P<0.001. Two-tailed Student’s unpaired t test analysis (A, B, C). Data are represented as mean ± SEM.

Supplementary figure 4. Losing weight through changing diet did not reverse the pathological manifestations of TMJ OA. (A) Animal experimental scheme. (B) Changes of the body weight of mice in each group during the experimental period (n=6). (C-D) Representative H&E staining images (C) and SO staining images (D) of the sagittal plane of the condyle. Scale bar, 100 µm. (E) The modified Mankin score of the cartilage (n=6). *** *P*<0.001; ns, *P* > 0.05. One-way ANOVA followed by Tukey’s multiple comparisons test (E). Data are represented as mean ± SEM.

Supplementary figure 5. Representative confocal images of DiO-labelled EVs in TMJ joint. The tissues were stained with DAPI (blue) for nuclei. Red arrowheads indicate the EVs. Scale bar, 100 µm.

Supplementary figure 6. Adipose tissue-derived EVs from obese mice inhibit the osteogenic function in vitro. (A) The representative images of ALP staining of MC3T3 cells after 7 days of osteogenic induction. Scale bar, 100 µm. (B) The representative images of ALP staining of BMSCs after 7 days of osteogenic induction. Scale bar, 100 µm. (C). Osteogenic mRNA expression of MC3T3 cells after 7 days of osteogenic induction (n=3). (D). Osteogenic mRNA expression of BMSCs after 7 days of osteogenic induction (n=3). * P<0.05; ** P<0.01; *** P<0.001; ns, P > 0.05. One-way ANOVA followed by Tukey’s multiple comparisons test. Data are represented as mean ± SEM.

Supplemental figure 7. Blocking EVs release by GW4869 could counteract the adverse effects of adipose tissue in obese mice on chondrocytes. (A) Experimental scheme for coculture. (B-C) The effect of adipose tissue co-culture on chondrocyte apoptosis was detected by flow cytometry and statistically analyzed (n=4). (D-E) The representative images showing the proliferation and migration ability of chondrocytes were obtained by the scratch test and statistically analyzed (n=4). Scale bar, 100 µm. (F-G) The protein levels of COL2, SOX9, and MMP13 in chondrocyte was detected by WB assay and statistically analyzed (n=4). * P<0.05; ** P<0.01; *** P<0.001; ns, P > 0.05. One-way ANOVA followed by Tukey’s multiple comparisons test (C, E, G). Data are represented as mean ± SEM.

Supplemental figure 8. Fundamental characteristics of adipose tissue and cartilage in cKO mice.

(A) Body weight, body length and representative photos of WT and cKO mice after 12 weeks of CD or HFD feeding (n=5-7). (B) The percentage of visceral adipose tissue weight relative to the body weight in WT and cKO mice after 12 weeks of CD or HFD feeding (n=5-7). (C) Representative H&E staining images of the adipose tissue in Ggpps cKO mice and their WT littermates fed with CD or HFD for 12 weeks. Scale bar, 100 µm.

* P<0.05; ** P<0.01; *** P<0.001; ns, P > 0.05. One-way ANOVA followed by Tukey’s multiple comparisons test (A, B). Data are represented as mean ± SEM.

Supplemental figure 9. Biocompatibility evaluation of the injection of EVs and antagomiR.

H&E staining images of the liver, spleen and kidney tissue from the mice with different treatments. Scale bar, 100 µm.
